# Optimizing Conditions in the Acid Tolerance Test for Potential Probiotics Using Response Surface Methodology

**DOI:** 10.1128/spectrum.01625-22

**Published:** 2022-07-25

**Authors:** Hye In Ko, Chang Hee Jeong, Sung Wook Hong, Jong-Bang Eun, Tae-Woon Kim

**Affiliations:** a Technology Innovation Research Division, World Institute of Kimchi, Gwangju, Republic of Korea; b Department of Integrative Food, Bioscience and Biotechnology, Chonnam National University, Gwangju, Republic of Korea; University of Minnesota

**Keywords:** acid tolerance test, probiotics, lactic acid bacteria, response surface methodology

## Abstract

Acid tolerance is an important feature of probiotic development. It is one of the factors underlying the beneficial effects of probiotics in the intestine. However, the methods used by different researchers to test acid tolerance vary, causing confusion in the interpretation of the results. Therefore, in this study, we determine the optimal conditions for the acid tolerance test using response surface methodology. The factors of pH (2.5 to 3.5), exposure time (1 to 2 h), and pepsin (presence or absence) were used as independent variables, and the survival rates of seven strains (Lacticaseibacillus casei KACC 12413, Lactiplantibacillus plantarum KACC 15357, Limosilactobacillus fermentum KACC 11441, *Lactiplantibacillus plantarum* WCFS1, Lacticaseibacillus rhamnosus GG, *Lactiplantibacillus plantarum* KCTC 21024, and *Lactiplantibacillus plantarum* WiKim 0112) known to have probiotic properties were used as dependent variables. The results of the analysis of variance (ANOVA) indicated that the pH value and exposure time in acidic environments significantly affected the acid tolerance test model, and their interaction also had an effect (*P* < 0.05). Using the ANOVA results, the condition of the acid tolerance test was optimized with a target of an 85% survival rate for each strain. The optimized conditions of the acid tolerance test were as follows: pH 2.92, exposure time of 1.73 h, and presence of pepsin and pH 3, exposure time of 1.98 h, and absence of pepsin. These results can optimize strain selection with rigorous acid tolerance without confusion by unifying the conditions for the acid tolerance test.

**IMPORTANCE** The acid tolerance test, which is the first step in selecting probiotics, is not standardized and can often cause confusion in the interpretation of results. Thus, in the present study, we optimized the conditions for the acid tolerance test using response surface methodology. These optimized conditions can be used to screen for strains with acid tolerance.

## INTRODUCTION

Lactic acid bacteria (LAB), including *Lactobacillus*, *Lactiplantibacillus*, *Lacticaseibacillus*, and *Limosilactobacillus*, are commonly found in fermented foods and are widely used strains in probiotics (1). Probiotic strains have been reported to exhibit various beneficial effects on human health, including antimicrobial, antidiabetic, antiobesity, antihypertensive, anticarcinogenic, and anticholesterol activities (2, 3). According to a previous study, Latilactobacillus sakei OK67 inhibited an increase in blood glucose levels, body weight gain, and lipopolysaccharide production from gut microbiota in mice fed a high-fat diet (4). In addition, Lacticaseibacillus casei ATCC 393 induces apoptosis in colon carcinoma cells (5). To confer health benefits on the host, probiotics need to reach the intestine through harsh gastrointestinal conditions such as low pH values, pepsin, bile, and proteolytic enzymes (6). In particular, the low-pH environment in gastric juice is the most important factor affecting the viability of probiotic candidate strains (7). Therefore, it is necessary to conduct an appropriate acid tolerance test for probiotic candidate strains.

In previous studies, acid tolerance tests of candidate probiotic strains were conducted under varied conditions (3, 8). Hence, the evaluation of acid tolerance of the same strain would sometimes have different results. For instance, Lacticaseibacillus rhamnosus GG (LGG) was exposed to pH 3 medium containing pepsin for 90 or 180 min to evaluate the acid tolerance of the cells (9). As a result, the number of LGG bacteria was decreased slightly to 5.86 ± 0.45 log CFU/mL at 90 min and 5.06 ± 0.12 log CFU/mL at 180 min of exposure compared to that of the control (6.22 ± 0.05 log CFU/mL). Contrastingly, in the study by Jung et al. (10), exposure of the same strain to pH 2.5 medium without pepsin for 2 h showed a remarkable decrease in the number of the cells (7.00 ± 0.67 log CFU/mL) compared to that of the control (9.79 ± 0.20 log CFU/mL). Thus, the method for conducting acid tolerance tests must be standardized and optimized to enhance the accuracy of the test. A previous study attempted to standardize the acid tolerance test method for probiotics (6); however, it was limited by the fact that only three strains were used for standardization and the interactions among independent factors were not considered.

Exposure time and pH are crucial characteristics affecting the survival rate of strains during acid tolerance tests (11). Furthermore, the presence of pepsin affects the survival of some strains (11). Indeed, the acid tolerance of probiotic candidate strains can be also affected by the interaction of various independent factors. Response surface methodology (RSM) is an effective mathematical and statistical tool for deriving an optimization model that reflects the influence of various factors (12). RSM, which is a multivariate technique, has been applied to optimize pharmaceuticals, food production, and biochemical conditions (12, 13). According to a previous study, RSM based on central composite design (CCD) was applied with independent variables such as glycerol, sodium glutamate, and skim milk to optimize the cryoprotective medium to increase the viability of Streptococcus thermophilus (13). Furthermore, it was applied to obtain independent variable ratios based on the interaction of pH, incubation time, soluble starch, and beef extract to optimize α-amylase production from Bacillus licheniformis WF67 (14). Similarly, RSM can be widely applied to determine the influence of these independent variables on the dependent variables and optimize the test conditions (15).

Thus, in this study, RSM based on the CCD approach was applied with pH value, incubation time, and pepsin presence as independent variables, and the survival rates of seven strains, which are known to have probiotic properties, as dependent variables to optimize the conditions of the acid tolerance test for probiotic candidate strains.

## RESULTS AND DISCUSSION

### Acid tolerance test of strains with probiotic properties.

**FIG 1:**
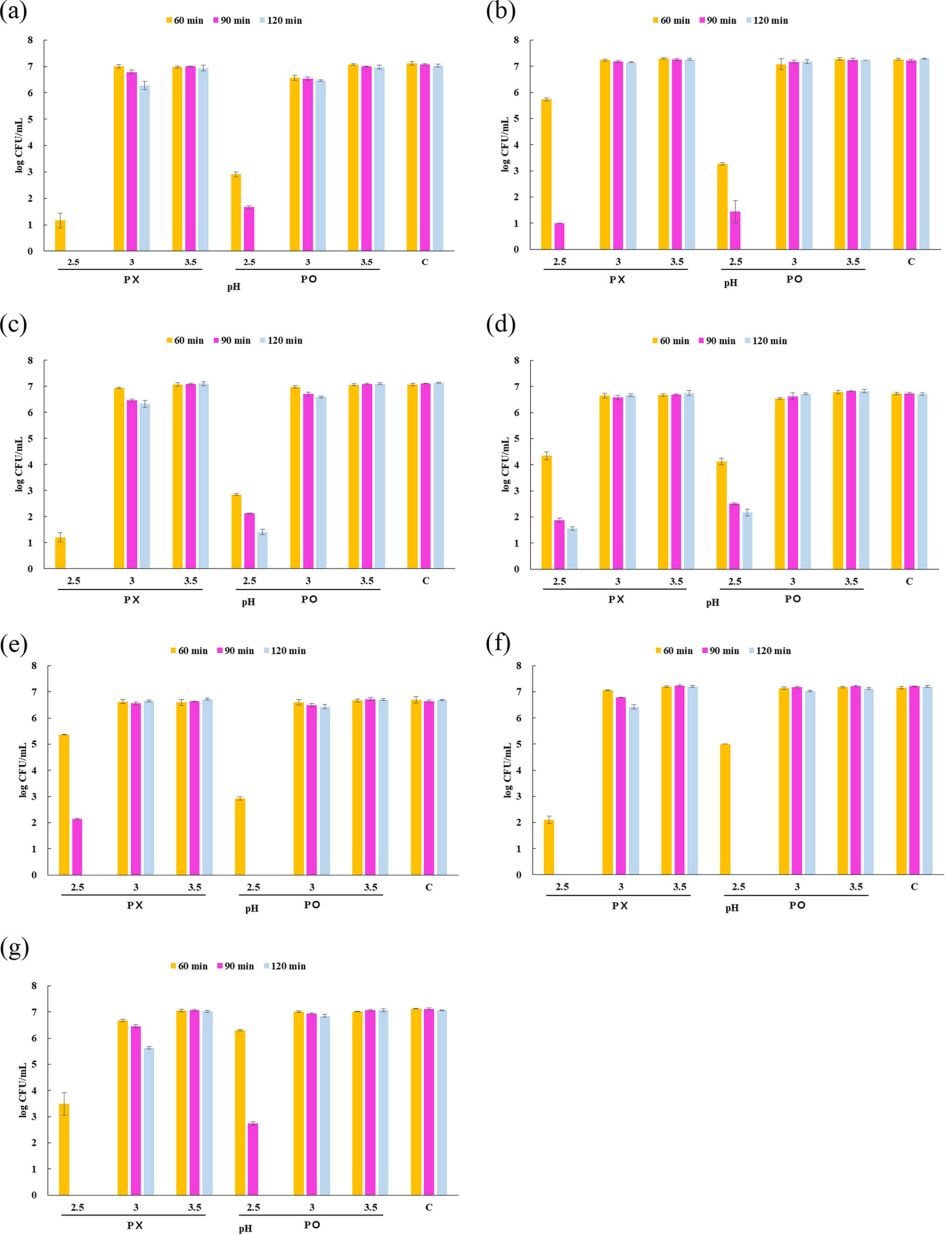
Survival of strains in the different acidic environments. (a) KCTC 21024 (*Lactiplantibacillus plantarum*); (b) KACC 15357 (*Lactiplantibacillus plantarum*); (c) WCFS1 (*Lactiplantibacillus plantarum* WCFS1); (d) LGG (*Lacticaseibacillus rhamnosus* GG); (e) KACC 12413 (*Lacticaseibacillus casei*); (f) KACC 11441 (*Limosilactobacillus fermentum*); (g) WiKim 0112 (*Lactiplantibacillus plantarum*). C, control; P×, no added pepsin; P○, added pepsin.

The results for cell viability under each condition are shown in Fig. 1. When exposed to simulated gastric juices (SGJs) at pH 2.5 for 60 min, the viability of most strains was low, 16 to 79%, except for KACC 12413 (presence of pepsin, 80.07%) and WiKim 0112 (presence of pepsin, 88.45%). When exposed to pH 2.5 SGJs and the absence of pepsin for 90 min, only KACC 12413 (32.30%), LGG (27.88%), and KACC 15357 (13.87%) survived, whereas when exposed to pH 2.5 SGJs and the presence of pepsin for 90 min, the viability of most strains was in the range of 19 to 39%, except for KACC 11441 and KACC 12413, which did not survive. After exposure to pH 2.5 SGJs for 120 min, only LGG (absence of pepsin, 23.26%; presence of pepsin, 32.39%) and WCFS1 (presence of pepsin, 19.89%) survived. After exposure to pH 3 SGJs for 120 min, the viability of all strains was in the range of 79 to 101%, which was higher than that when exposed to pH 2.5 SGJs. After exposure to pH 3.5 SGJs for 120 min, the viability of all strains was the highest, ranging from 98% to 102%. In our study, each strain showed a low survival rate of less than 70% when exposed to SGJs at pH 2.5 to 3 for 2 to 6 h (data not shown).

Lactic acid bacterium strains exhibited various acid tolerance strategies. This includes production of alkaline substances through the arginine dihydrolase system to neutralize acid, neutralization of protons in carbon dioxide produced by malolactic fermentation, and transport of protons by activation of proton pumps such as F_1_-F_0_-ATPase (16). In our results, the viability of most strains showed a tendency to decrease as the pH decreased and exposure time increased. At pH 2.5, cell viability decreased more rapidly as the exposure time increased than at pH 3. Interestingly, pepsin exhibited different effects on cell viability, depending on the strain. Pepsin is known to decrease the viability of microorganisms via proteolytic activity (17). However, the viability of KACC 21024, WCFS1, LGG, KACC 11441, and WiKim 0112 cells was increased by exposure to pepsin (Fig. 1). This result is similar to that of a previous study in which the viability of Bifidobacterium animalis subspecies increased when exposed to pepsin. Although the mechanisms underlying pepsin’s ability to enhance acid tolerance of lactic acid bacteria have not been elucidated completely, a previous study hypothesized that pepsin might help to maintain pH homeostasis by supporting the role of H^+^-ATPase in Bifidobacterium animalis subsp. *lactis* (18). This can be attributed to pepsin enhancing the action of the proton pump through ATP production (18). This hypothesis remains unconfirmed, although our results were also postulated for similar reasons.

Additionally, most of the strains used in this study showed high rates of survival when exposed to SGJ prepared with MRS broth for 2 h, unlike SGJ prepared with sterile saline (Fig. 1; see also Table S1 in the supplemental material). The increase of survival rate for LAB in SGJ with MRS broth is presumably due to the abundant nutrients present in MRS broth, so SGJ with MRS may not be appropriate to accurately select strains with acid tolerance (19). However, SGJ with sterile saline, the condition used to optimize the acid tolerance test in this study, provides a harsher environment for microorganisms, which can be a rigorous standard to select bacteria with acid tolerance.

### Experimental design and analysis for optimization.

The experimental design used to optimize the conditions of the acid tolerance test is presented in Table 1. The pH value, exposure time, and presence of pepsin were independent variables, and the survival rate of each strain was a dependent variable. Statistical analyses were performed on the basis of these variables. A quadratic regression equation was used to calculate the interactions among the factors. The formula for the factors was expressed according to the following equations:

**TABLE 1:** Central composite design for optimization of acid tolerance test

Run	Independent variable			Dependent variable (%)						
	pH	Time (h)	Pepsin	KCTC 21024^*a*^	KACC 15357^*a*^	WCFS1^*b*^	LGG^*c*^	KACC 12413^*d*^	KACC 11441^*e*^	WiKim 0112^*a*^
1	3	1	Added	92.412	97.384	98.789	97.126	98.645	99.843	98.466
2	3.5	1.5	Added	98.858	100.503	99.639	101.260	101.162	100.040	99.283
3	3.5	2	Added	99.174	100.501	99.518	101.770	100.212	98.935	100.000
4	2.5	1	Not added	16.305	78.914	16.981	64.596	80.069	29.344	48.892
5	3	1	Not added	98.512	99.548	98.204	98.787	98.956	98.647	93.743
6	3.5	1	Not added	98.162	100.418	99.968	99.144	98.501	100.605	98.952
7	3	1.5	Not added	95.840	99.482	90.818	97.617	98.861	94.086	90.726
8	3	2	Added	91.917	98.459	92.453	100.133	96.185	97.600	97.029
9	3	1.5	Not added	95.840	99.482	90.818	97.617	98.861	94.086	90.726
10	3.5	1	Added	99.494	100.110	98.789	100.842	99.617	100.301	98.537
11	3	1.5	Not added	95.840	99.482	90.818	97.617	98.861	94.086	90.726
12	3.5	2	Not added	98.683	99.705	99.587	100.6110	100.466	99.951	99.470
13	2.5	2	Not added	0	0	0	23.260	0	0	0
14	3	2	Not added	89.362	98.111	88.747	99.410	99.435	89.106	79.652
15	3	1.5	Added	92.298	99.479	94.321	98.353	97.664	99.519	97.539
16	3	1.5	Not added	95.840	99.482	90.818	97.617	98.861	94.086	90.726
17	2.5	1.5	Not added	0	13.872	0	27.876	32.298	0	0
18	2.5	2	Added	0	0	19.888	32.389	0	0	0
19	3	1.5	Added	92.298	99.479	94.321	98.353	97.664	99.519	97.539
20	3	1.5	Not added	95.840	99.482	90.818	97.617	98.861	94.086	90.726
21	3	1.5	Added	92.298	99.479	94.321	98.353	97.664	99.519	97.539
22	3	1.5	Added	92.298	99.479	94.321	98.353	97.664	99.519	97.539
23	3	1.5	Added	92.298	99.479	94.321	98.353	97.664	99.519	97.539
24	2.5	1.5	Added	23.548	19.986	29.871	37.221	0	0	38.519
25	2.5	1	Added	40.907	44.928	40.340	61.316	43.802	69.883	88.451
26	3.5	1.5	Not added	98.837	100.522	99.754	99.219	99.809	100.377	99.266

a *Lactiplantibacillus plantarum*. To over 100% means that it was not inhibited.

b *Lactiplantibacillus plantarum* WCFS1.

c *Lacticaseibacillus rhamnosus* GG.

d *Lacticaseibacillus casei*.

e *Limosilactobacillus fermentum*.

Survival rate of KCTC 21024 = 93.75 + 42.70*A* − 5.55*B* − 1.11*C* + 7.18*AB* + 3.86*AC* + 1.40*BC* − 37.65*A*^2^ + 0.091*B*^2^

Survival rate of KACC 15357 = 98.38 + 37.00*A* − 10.38*B* + 1.12*C* + 15.44*AB* − 2.36*AC* − 3.13*BC* − 36.92*A*^2^ + 2.74*B*^2^

Survival rate of WCFS1 = 92.56 + 40.85*A* − 4.41*B* − 3.60*C* + 4.72*AB* + 6.21*AC* − 0.063*BC* − 35.21*A*^2^ + 2.02*B*^2^

Survival rate of LGG = 97.20 + 29.68*A* − 5.35*B* − 0.88*C* + 9.08*AB* + 0.86*AC* – 1.19*BC* − 28.83*A*^2^ + 3.64*B*^2^

Survival rate of KACC 12413 = 97.30 + 36.97*A* − 10.27*B* + 2.92*C* + 15.80*AB* – 5.90*AC* − 2.66*BC* − 36.58*A*^2^ + 3.41*B*^2^

Survival rate of KACC 11441 = 95.04 + 41.75*A* − 9.42*B* − 2.91*C* + 12.15*AB* + 3.52*AC* + 2.83*BC* − 40.53*A*^2^ + 5.66*B*^2^

**FIG 2:**
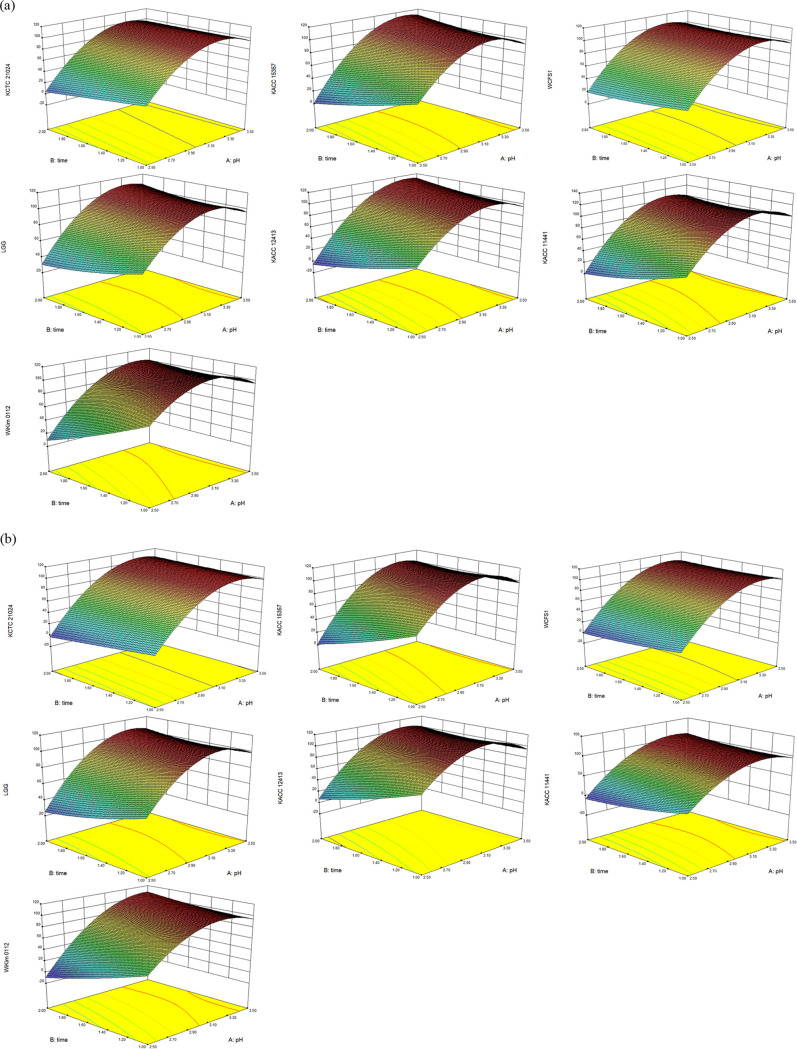
3D surface plots for survival rate of strains in different acidic environments. (a) Added pepsin; (b) no added pepsin. KCTC 21024 and KACC 15357, *Lactiplantibacillus plantarum*; WCFS1, *Lactiplantibacillus plantarum* WCFS1; LGG, *Lacticaseibacillus rhamnosus* GG; KACC 12413, *Lacticaseibacillus casei*; KACC 11441, *Limosilactobacillus fermentum*; WiKim 0112, *Lactiplantibacillus plantarum*.

Survival rate of WiKim 0112 = 92.83 + 34.97*A* − 12.57*B* − 5.17*C* + 17.42*AB* + 6.50*AC* + 2.16*BC* − 30.31*A*^2^ + 2.64*B*^2^

where *A* is the pH, *B* is the exposure time, and *C* is the presence of pepsin. Analysis of variance (ANOVA) was applied to confirm the goodness of fit of this model and the interaction of the factors statistically. The results are presented in Table 2 and Table S2. Further, in Fig. 2, three-dimensional (3D) response surface plots related to variables are visualized to confirm the interaction of the factors. All the models in Table 2 had statistically significant effects on each dependent variable (*P* < 0.05). The results in Table 2 show that pH and pH^2^ significantly influenced the survival rates of KCTC 21024 and KACC 11441 (*P* < 0.0001). The pH, interaction of pH and time, and pH^2^ significance affected the survival rate of KACC 15357, LGG, KACC 12413, and WiKim 0112 (*P* < 0.0001). In addition, pH, pepsin, interaction of pH and pepsin, and pH^2^ significantly influenced the survival rate of WCFS1 (*P* < 0.0001). Moreover, the *R*^2^ and adjusted *R*^2^ coefficients in all models exceeded 0.9, indicating that the reliability of this model was satisfactory (20). The *F* value is used to evaluate the influence of parameters on the model; a high *F* value means that the parameter has a large influence on the model (20). According to the *F* value, the most influential parameter in KCTC 20104 was pH, followed by pH^2^ and exposure time. The most influential parameters in KACC 15357, LGG, KACC 12413, and WiKim 0112 were pH, followed by pH^2^ and interaction of pH and exposure time. The most influential parameter in WCFS1 was pH, followed by pH^2^ and interaction of pH and pepsin. In addition, the most influential parameter in KACC 11441 was pH, followed by pH^2^. These results showed that each independent variable can influence the acid tolerance of strains, and their interactions can also influence the acid tolerance test of strains. Therefore, unlike the previous study, which considered only the influence of each independent factor on the dependent factor, these results statistically offered the influence of the interaction of independent factors on dependent factors. Hence, these experimental models can be used to forecast the optimum conditions for acid tolerance tests.

**TABLE 2:** ANOVA result for response surface model

Strain and parameter	Model	*A*-pH	*B*-time	*C*-pepsin	*AB* ^*h*^	*AC* ^*h*^	*BC* ^*h*^	*A* ^2^ ^*i*^	*B* ^2^ ^*i*^	Residual	Lack of fit	Pure error	Cor total^*j*^
KCTC 21024^*a*^													
Sum of squares	32,041.58	21,883.5	370.255	31.77028	412.028	178.695	23.48756	7,829.55	0.046038	470.9227	470.9227	0	32,512.5
df^*f*^	8	1	1	1	1	1	1	1	1	17	9	8	25
Mean square	4,005.197	21,883.5	370.255	31.77028	412.028	178.695	23.48756	7,829.55	0.046038	27.70133	52.32474	0	
*F* value	144.585	789.9802	13.36596	1.146886	14.87394	6.450771	0.847886	282.6416	0.001662				
*P* value^*g*^	<0.0001	<0.0001	0.002	0.2992	0.0013	0.0211	0.37	<0.0001	0.968				
KACC 15357^*a*^													
Sum of squares	28,207.52	16,432.2	1,292.227	32.87791	1,907.172	66.93266	117.8267	7,529.076	41.35426	1,241.048	1,241.048	0	29,448.57
df^*f*^	8	1	1	1	1	1	1	1	1	17	9	8	25
Mean square	3,525.94	16,432.2	1,292.227	32.87791	1,907.172	66.93266	117.8267	7,529.076	41.35426	73.0028	137.8942	0	
*F* value	48.2987	225.09	17.70106	0.450365	26.12464	0.916851	1.614002	103.1341	0.566475				
*P* value^*g*^	<0.0001	<0.0001	0.0006	0.5112	<0.0001	0.3517	0.221	<0.0001	0.462				
WCFS1^*b*^													
Sum of squares	28,919.72	20,022.56	233.0045	336.6783	178.4407	462.2972	0.04808	6,848.207	22.58317	171.4932	171.4932	0	29,091.21
df^*f*^	8	1	1	1	1	1	1	1	1	17	9	8	25
Mean square	3,614.965	20,022.56	233.0045	336.6783	178.4407	462.2972	0.04808	6,848.207	22.58317	10.08784	19.05481	0	
*F* value	358.3488	1,984.821	23.09757	33.37467	17.6887	45.82718	0.004766	678.8577	2.238653				
*P* value^*g*^	<0.0001	<0.0001	0.0002	<0.0001	0.0006	<0.0001	0.9458	<0.0001	0.1529				
LGG^*c*^													
Sum of squares	16,562.02	10,572.54	343.8762	20.05882	659.9132	8.831821	16.93154	4,591.399	73.16354	436.3734	436.3734	0	16,998.4
df^*f*^	8	1	1	1	1	1	1	1	1	17	9	8	25
Mean square	2,070.253	10,572.54	343.8762	20.05882	659.9132	8.831821	16.93154	4,591.399	73.16354	25.66902	48.48593	0	
*F* value	80.6518	411.8795	13.39655	0.781441	25.70854	0.344065	0.65961	178.8693	2.850266				
*P* value^*g*^	<0.0001	<0.0001	0.0019	0.389	<0.0001	0.5652	0.4279	<0.0001	0.1096				
KACC 12413^*d*^													
Sum of squares	28,493.96	16,398.14	1,266.74	221.5218	1,998.142	417.4936	85.10509	7,390.946	64.19943	1,268.809	1,268.809	0	29,762.77
df^*f*^	8	1	1	1	1	1	1	1	1	17	9	8	25
Mean square	3,561.746	16,398.14	1,266.74	221.5218	1,998.142	417.4936	85.10509	7,390.946	64.19943	74.63583	140.9788	0	
*F* value	47.72166	219.7087	16.97227	2.968036	26.77188	5.593742	1.140271	99.02679	0.860169				
*P* value^*g*^	<0.0001	<0.0001	0.0007	0.1031	<0.0001	0.0302	0.3005	<0.0001	0.3667				
KACC 11441^*e*^													
Sum of squares	33,317.49	20,915.28	1,064.655	220.6086	1,181.168	148.37	96.0676	9,073.873	177.2401	1,363.3	1,363.3	0	34,680.79
df^*f*^	8	1	1	1	1	1	1	1	1	17	9	8	25
Mean square	4,164.686	20,915.28	1,064.655	220.6086	1,181.168	148.37	96.0676	9,073.873	177.2401	80.19409	151.4777	0	
*F* value	51.93258	260.8082	13.27598	2.750934	14.72886	1.850136	1.197939	113.1489	2.210139				
*P* value^*g*^	<0.0001	<0.0001	0.002	0.1155	0.0013	0.1915	0.289	<0.0001	0.1554				
WiKim 0112^*a*^													
Sum of squares	25,843.87	14,675.23	1,897.285	694.5063	2,426.423	506.2875	56.15729	5,075.585	38.57552	1,273.981	1,273.981	0	27,117.85
df^*f*^	8	1	1	1	1	1	1	1	1	17	9	8	25
Mean square	3,230.484	14,675.23	1,897.285	694.5063	2,426.423	506.2875	56.15729	5,075.585	38.57552	74.94008	141.5535	0	
*F* value	43.10756	195.8261	25.31737	9.267489	32.37817	6.755898	0.749363	67.72859	0.514751				
*P* value^*g*^	<0.0001	<0.0001	0.0001	0.0073	<0.0001	0.0187	0.3987	<0.0001	0.4828				

a *Lactiplantibacillus plantarum*.

b *Lactiplantibacillus plantarum* WCFS1.

c *Lacticaseibacillus rhamnosus* GG.

d *Lacticaseibacillus casei*.

e *Limosilactobacillus fermentum*.

f df, degree of freedom.

g *P* value of <0.05: model at 95% confidence level.

h Variable interaction effects.

i Second-order effects.

j Sum of squares total corrected for the mean.

### Optimization and validation of acid tolerance test.

The conditions of the acid tolerance test were optimized by analysis of the ANOVA results. The criteria for cell viability, pH, exposure time, and presence of pepsin are listed in Table S3. A cell viability of 80% or more was used as the criterion for a highly acid-tolerant strain (1). The criteria were set such that the range of the strain survival rate was 80 to 95%, and the target was set at 85%. The optimum conditions for the acid tolerance test, based on these criteria, are listed in Table 3. The results showed that the optimum pH value and exposure time varied depending on the presence or absence of pepsin. The acid tolerance test with pepsin can be applied to the *in vitro* test of probiotics that must pass through the gastric phase. The acid tolerance test without pepsin can be applied to investigate the acid tolerance of strains as starter cultures in fermented products, such as fermented juices with low pH (21). Accordingly, in the presence of pepsin, a pH of 2.92 and an exposure time of 1.73 h (test 1) and, in the absence of pepsin, a pH of 3 and an exposure time of 1.98 h (test 2) were determined.

**TABLE 3:** Optimal conditions for acid tolerance test expected in RSM

Test	pH	Time (h)	Pepsin	Predicted value (%) for strain:						
				KCTC 21024^*a*^	KACC 15357^*a*^	WCFS1^*b*^	LGG^*c*^	KACC 12413^*d*^	KACC 11441^*e*^	WiKim 0112^*a*^
1	2.92	1.73	Added	83.7336	85.8647	87.5668	90.7085	82.3648	85.2224	85.0001
2	3.00	1.98	Not added	89.0169	89.3203	86.7927	93.5905	91.184	91.3036	80.4062

a *Lactiplantibacillus plantarum*.

b *Lactiplantibacillus plantarum* WCFS1.

c *Lacticaseibacillus rhamnosus* GG.

d *Lacticaseibacillus casei*.

e *Limosilactobacillus fermentum*.

To confirm the effectiveness of the conditions in the acid tolerance test based on CCD, an optimized acid tolerance test was performed using 18 strains (Table 4). The survival rates of LGG, KCKM 245, KCKM 429, KCKM 438, KCKM 597, KCKM 625, KCKM 720, KCKM 729, KCKM 851, KCKM 991, KCKM 998, KCKM 1014, 1086, KCKM 1105, and KCKM 469 in test 2 were high (>80%), whereas those of KCKM 10 and KCKM 12 in tests 1 and 2 and KCKM 469 in test 1 were significantly low. Leuconostoc mesenteroides is the predominant bacterium in the initial and middle phases of kimchi fermentation (approximate pH of 5), and the number of this strain decreases as pH decreases during kimchi fermentation (22, 23). Therefore, Leuconostoc mesenteroides is believed to have weak acid tolerance, which is consistent with the acid tolerance results of KCKM 10 and KCKM 12. These results indicate that strains with or without acid tolerance could be precisely sorted by our optimized conditions in the acid tolerance test.

**TABLE 4:** Survival rate of a variety of strains under optimized acid tolerance test conditions

Strain	Survival rate (%)		Independent-sample *t* test	
	Test 1	Test 2	*t* value	*P* value
LGG^*a*^	99.97 ± 0.43	99.68 ± 0.21	1.039	0.358
KCKM 10^*b*^	0.00 ± 0.00	42.84 ± 1.33	−55.683	0.000
KCKM 12^*b*^	0.00 ± 0.00	71.02 ± 0.41	−296.935	0.000
KCKM 245^*c*^	102.25 ± 0.65	100.02 ± 0.80	3.760	0.020
KCKM 429^*d*^	94.30 ± 1.09	99.12 ± 0.27	−7.430	0.002
KCKM 438^*e*^	98.46 ± 0.93	98.46 ± 1.47	0.003	0.998
KCKM 469^*f*^	71.95 ± 2.33	99.07 ± 1.51	−16.893	0.000
KCKM 597^*d*^	99.44 ± 0.22	99.65 ± 0.49	−0.669	0.540
KCKM 625^*c*^	95.32 ± 0.63	96.79 ± 0.39	−3.438	0.026
KCKM 720^*d*^	97.18 ± 0.15	99.17 ± 0.54	−6.194	0.003
KCKM 729^*g*^	96.35 ± 0.52	99.52 ± 1.22	−4.143	0.014
KCKM 851^*e*^	84.03 ± 0.89	96.85 ± 1.44	−13.150	0.000
KCKM 990^*d*^	102.35 ± 0.31	102.04 ± 0.15	1.574	0.191
KCKM 991^*h*^	98.82 ± 0.53	98.86 ± 0.68	−0.075	0.944
KCKM 998^*g*^	99.30 ± 0.72	98.03 ± 0.79	2.056	0.110
KCKM 1014^*c*^	89.22 ± 9.27	99.02 ± 0.60	−1.829	0.208
KCKM 1086^*i*^	87.25 ± 0.39	98.29 ± 0.43	−33.100	0.000
KCKM 1105^*i*^	88.84 ± 0.91	90.15 ± 1.64	−1.217	0.291

a *Lacticaseibacillus rhamnosus* GG.

b Leuconostoc mesenteroides.

c *Lacticaseibacillus paracasei*. To over 100% means that it was not inhibited.

d *Lactiplantibacillus plantarum*.

e Lactococcus lactis.

f Enterococcus faecium.

g *Limosilactobacillus fermentum*.

h *Lacticaseibacillus casei*.

i *Lactiplantibacillus paraplantarum*.

Based on the independent-sample *t* test, KCKM 10, KCKM 12, KCKM 245, KCKM 429, KCKM 469, KCKM 625, KCKM 720, KCKM 729, KCKM 851, and KCKM 1086 exhibited significant differences between test results (*P* < 0.05). Even though the difference of the pH between two tests was only 0.08, the results varied depending on the strain. These results indicate that optimized tests can be used differently, depending on the purpose.

In this study, we optimized the conditions for the acid tolerance test by applying RSM based on the CCD. The optimized conditions were as follows: pH 2.92 and exposure time of 1.73 h in the presence of pepsin or pH 3 and exposure time of 1.98 h in the absence of pepsin. These conditions were effective in accurately selecting a strain with acid tolerance. Each condition can be employed to confirm acid tolerance in SGJ with pepsin and in a low-pH environment without pepsin. However, SGJ supplemented with pepsin has the limitation of not being able to completely reproduce the dynamic gastric environment. Therefore, this condition can be employed to confirm acid tolerance of probiotic candidates before *in vivo* study. Furthermore, our results can be suggested as a method to select a strain with acid tolerance rigorously by optimizing the conditions of the acid tolerance test.

## MATERIALS AND METHODS

### LAB strains and sample collection.

Seven strains with probiotic properties were used to optimize the acid tolerance test method (Table 5). *Lacticaseibacillus casei* KACC 12413 (ATCC 393), *Lactiplantibacillus plantarum* KACC 15357, and Limosilactobacillus fermentum KACC 11441 (ATCC 14931) were provided by the Korean Agricultural Culture Collection (KACC; Wanju, South Korea), *Lactiplantibacillus plantarum* WCFS1 (ATCC BAA-793), LGG (ATCC 53103), and *Lactiplantibacillus plantarum* KCTC 21024 (ATCC 8014) were obtained from the Korean Collection for Type Cultures (KCTC; Jeongeup, South Korea). *Lactiplantibacillus plantarum* WiKim 0112 was isolated from kimchi. In addition, Leuconostoc mesenteroides KCKM 10, Leuconostoc mesenteroides KCKM 12, Lacticaseibacillus paracasei KCKM 245, *Lactiplantibacillus plantarum* KCKM 429, Lactococcus lactis KCKM 438, Enterococcus faecium KCKM 469, *Lactiplantibacillus plantarum* KCKM 597, *Lacticaseibacillus paracasei* KCKM 625, *Lactiplantibacillus plantarum* KCKM 720, *Limosilactobacillus fermentum* KCKM 729, Lactococcus lactis KCKM 851, *Lactiplantibacillus plantarum* KCKM 990, *Lacticaseibacillus casei* KCKM 991, *Limosilactobacillus fermentum* KCKM 998, *Lacticaseibacillus paracasei* KCKM 1014, Lactiplantibacillus paraplantarum KCKM 1086, and *Lactiplantibacillus paraplantarum* KCKM 1105 were isolated from kimchi provided by the Korean Collection for Kimchi Microorganisms (KCKM; Gwangju, South Korea) and used for acid tolerance tests.

**TABLE 5:** Lactic acid bacteria used in this study and their acid tolerance

Strain	Source	Survival (log CFU/mL)^*a*^		Reference
		Control	Acidic stress	
*Lactiplantibacillus plantarum*	KCTC 21024 (ATCC 8014)	8.24	5.94	26
*Lactiplantibacillus plantarum*	KACC 15357			http://genebank.rda.go.kr/microbeSearchView.do?sFlag=ONE&sStrainsn=31018
*Lactiplantibacillus plantarum* WCFS1	ATCC BAA-793	6.39	4.43	9
*Lacticaseibacillus rhamnosus* GG	ATCC 53103	6.22	5.86	9
*Lacticaseibacillus casei*	KACC 12413 (ATCC 393)	7.45	4.96	27
*Limosilactobacillus fermentum*	KACC 11441 (ATCC 14931)			28
*Lactiplantibacillus plantarum*	WiKim 0112	9.22–9.29	8.28	24

a Conditions for the acid tolerance test are based on the papers referenced.

Strains were cultured in de Man, Rogosa, and Sharpe (MRS) broth at 37°C for 18 h. All cultures were maintained with skim milk at −80°C and subcultured twice in MRS broth before the experiment.

### Experimental design and statistical analysis.

To optimize the acid tolerance test method, Design-Expert software (version 8.0.6, Stat-Ease, Inc., Minneapolis, MN, USA) was used for the experimental design using a central composite design and the optimization of the acid tolerance method. The pH, exposure time, and presence of pepsin were applied as independent variables, and the survival rate in the acidic environment of the seven strains was determined as the dependent variable. Table 6 lists the independent variables and levels. To predict the optimal conditions, the quadratic model was described by the following equation:

$$Y={\text{β}}_{0}+\overset{K}{\underset{i=1}{\sum }}{\text{β}}_{i}X_{i}+\overset{K}{\underset{i=1}{\sum }}{\text{β}}_{ii}X_{i}^{2}+\overset{K-1}{\underset{i=1}{\sum }}\overset{K}{\underset{j=1+1}{\sum }}{\text{β}}_{ij}X_{i}X_{j}+\text{ε}$$

where β_0_ is the model constant, β*_i_X_i_* is the linear term, $\beta_{ii}X_{i}^{2}$ is the quadratic term, and β*_ij_X_i_X_j_* is the two-factor interaction. Analysis of variance (ANOVA) was used to analyze the data and explain the interaction between variables with a 95% confidence level.

**TABLE 6:** Range and levels of continuous and categorical variables on RSM

Variable	Level		
	−Alpha (−1)	Middle (0)	+Alpha (+1)
pH	2	2.5	3
Time (h)	1	1.5	2
Pepsin	Added		Not added

### Preparation of SGJ.

Simulated gastric juices (SGJs) were prepared by adding pepsin from porcine (Sigma-Aldrich, St. Louis, MO, USA) to achieve 2,000 U/mL in 0.85% sterile saline, and the pH was adjusted to 2.5, 3, or 3.5, with 1 N hydrochloric acid (Daejung Chemicals & Metals Co., Ltd., Siheung, South Korea). SGJ was sterilized by filtering using a 0.22-μm filter membrane (Minisart NML-Sartorius, Göttingen, Germany). Sterile saline (pH 7) was used as the control. The range of pH was set to 2.5 to 3.5 because the pH of ingested food is known as pH 3, and the exposure time was set to 1 to 2 h because the recommended time of the gastric phase was 2 h (24, 25). The amount of enzyme was determined based on the method described by Minekus et al. (25). All the digestive juices were prepared prior to testing.

### Preparation of strains.

All strains used in this experiment were subcultured in MRS broth and incubated at 37°C for 18 h. All cultures were centrifuged at 10,000 × *g* for 5 min, and the cells were washed twice using 0.85% sterile saline.

### Acid tolerance test of strains.

The cells (1 × 10^7^ CFU/mL) were inoculated into six SGJs (pH 2.5, added pepsin; pH 2.5, no added pepsin; pH 3, added pepsin; pH 3, no added pepsin; pH 3.5, added pepsin; pH 3.5, no added pepsin) and control. The SGJs were incubated at 37°C for 60, 90, or 120 min. To determine the number of variable counts, SGJs were diluted 10-fold and plated on 3M Petrifilm lactic acid bacterial count plates (3M Co., St. Paul, MN, USA). Further, the lactic acid bacterial count plates were incubated at 37°C for 48 h, and the survival rate of the strains was calculated as described above using the following expression: survival rate (%) = log treatment CFU per mL/log control CFU per mL.

### Statistical analysis.

Each test was performed three times. To confirm the optimized test, an independent-sample *t* test was performed using SPSS 19 software (IBM, Chicago, IL, USA).
