# Supplementary material for: Optimizing Conditions in the Acid Tolerance Test for Potential Probiotics Using Response Surface Methodology
Source: Microbiol Spectr. 2022 Jul 25;10(4):e01625-22. doi: 10.1128/spectrum.01625-22 (PMC9430379; doi:10.1128/spectrum.01625-22)
Supplement: Supplemental file 1 — Supplemental material. Download spectrum.01625-22-s0001.pdf, PDF file, 0.2 MB [file spectrum.01625-22-s0001.pdf]

Table S1. Survival rate of each strain exposured SGJ with MRS (%).

Time (min)	KCTC 21024[a]		KACC 15357[a]		WCFS 1[b]		LGG[c]		KACC 12413[d]		KACC 11441[e]		WiKim 0112[a]	
	pH 2.5	pH 3	pH 2.5	pH 3	pH 2.5	pH 3	pH 2.5	pH 3	pH 2.5	pH 3	pH 2.5	pH 3	pH 2.5	pH 3
60	91.39	92.35	93.19	93.26	91.36	91.91	87.67	96.38	51.05	94.04	98.12	99.29	88.69	89.42
120	100.51	101.25	89.34	91.82	83.61	89.29	50.39	94.23	14.26	92.67	78.39	94.62	89.42	89.71

[a] *Lactiplantibacillus plantarum*.

[b] *Lactiplantibacillus plantarum* WCFS1.

[c] *Lacticaseibacillus rhamnosus* GG.

[d] *Lacticaseibacillus casei*.

[e] *Limosilactobacillus fermentum*.

Table S2. Details of ANOVA result for response surface model.

	KCTC 21024[a]	KACC 15357[a]	WCFS 1[b]	LGG[c]	KACC 12413[d]	KACC 11441[e]	WiKim 0112[a]
Std. Dev[f]	5.26	8.54	3.18	5.07	8.64	8.96	8.66
Mean	76.42	82.61	77.24	85.57	81.99	78.95	80.06
C.V.%[g]	6.89	10.34	4.11	5.92	10.54	11.34	10.81
R^{2h}	0.99	0.96	0.99	0.97	0.96	0.96	0.95
Adjusted R^2	0.98	0.94	0.99	0.96	0.94	0.94	0.93
Predicted R^2	0.95	0.83	0.98	0.90	0.83	0.84	0.81
Adeq precision[i]	34.71	22.82	55.42	27.40	23.93	23.27	24.48

[a] *Lactiplantibacillus plantarum*.

[b] *Lactiplantibacillus plantarum* WCFS1.

[c] *Lacticaseibacillus rhamnosus* GG.

[d] *Lacticaseibacillus casei*.

[e] *Limosilactobacillus fermentum*.

[f] standard deviation.

[g] coefficient variation.

[h] coefficient determination.

[i] adequate precision.

Table S3. Criteria for optimization of acid tolerance test.

Criteria	pH	Time (h)	Pepsin	Survival rate (%)						
				KCTC 21024[a]	KACC 15357[a]	WCFS 1[b]	LGG[c]	KACC 12413[d]	KACC 11441[e]	WiKim 0112[a]
In range	2.5-3.5	1-2	Added	80-95	80-95	80-95	80-95	80-95	80-95	80-95
Target	-	-	Not added	85	85	85	85	85	85	85

[a] *Lactiplantibacillus plantarum*.

[b] *Lactiplantibacillus plantarum* WCFS1.

[c] *Lacticaseibacillus rhamnosus* GG.

[d] *Lacticaseibacillus casei*.

[e] *Limosilactobacillus fermentum*.
